# Laparoscopic Long Mesh Surgery with Augmented Round Ligaments: A Novel Uterine Preservation Procedure For Apical Pelvic Organ Prolapse

**DOI:** 10.1038/s41598-020-63725-x

**Published:** 2020-04-20

**Authors:** Cheng-Yu Long, Chiu-Lin Wang, Kun-Ling Lin, Chin-Ru Ker, Zixi Loo, Yiyin Liu, Pei-Chi Wu

**Affiliations:** 1 Department of Obstetrics and Gynecology, Kaohsiung Municipal Siaogang Hospital, Kaohsiung Medical University, Kaohsiung, Taiwan; 2 Department of Obstetrics and Gynecology, Kaohsiung Medical University Hospital, Kaohsiung Medical University, Kaohsiung, Taiwan; 3 Department of Obstetrics and Gynecology, Kaohsiung Municipal Ta-Tung Hospital, Kaohsiung Medical University, Kaohsiung, Taiwan; 4 Department of Obstetrics and Gynecology, National Taiwan University Hospital, Taipei, Taiwan

**Keywords:** Urogenital diseases, Outcomes research

## Abstract

We aim to assess the surgical outcomes of our novel hysteropexy procedure, laparoscopic long mesh surgery (LLMS) with augmented round ligaments. Twenty-five consecutive women with stage II or greater main uterine prolapse defined by the POP quantification staging system were referred for LLMS. Long mesh is a synthetic T-shaped mesh, with the body fixed at the uterine cervix and the two arms fixed along the bilateral round ligaments. The clinical evaluations performed before and 6 months after surgery included pelvic examinations, urodynamic studies, and questionnaires for urinary and sexual symptoms. After a follow-up time of 12 to 24 months, the anatomical reduction rate was 92% (23/25) for the apical compartment. The average operative time was 65.4  ±  28.8 minutes. No major complications were recognized during LLMS. The lower urinary tract symptoms and scores on the questionnaires improved significantly after the surgery, except urgency urinary incontinence and nocturia. Neither voiding nor storage dysfunction was observed after the operations. All of the domains and total Female Sexual Function Index (FSFI) scores of the 15 sexually active women did not differ significantly after LLMS. The results of our study suggest that LLMS is an effective, safe, and time-saving hysteropexy surgery for the treatment of apical prolapse.

## Introduction

Pelvic organ prolapse (POP) refers to the downward displacement of pelvic organs^1^. The lifetime risk of surgery for POP in the general female population is 19%^1^. Uterine prolapse, defined by insufficient apical support, is an important issue in the POP field. An increasing number of women with POP choose not to have a hysterectomy for reasons of personal identity, perceived body image, or childbearing potential^2,3^.

We started to perform laparoscopic organopexy with non-mesh genital (LONG) suspension in 2014, as an innovative method for treating uterine prolapse in women who wish to preserve their uteri^4^. The LONG procedure has demonstrated promising preliminary results with a cohort of 40 women and a follow-up period of 12–30 months. The anatomical success rate was 85% with no concerning complications reported^4^.

Despite the favorable outcome, some skepticism has been raised regarding the durability of suspension power based solely on the adhesion between the transverse fascia beneath the rectus abdominis muscle and the uterus. Other concerns include the long-term effects of the untreated anterior or posterior compartment and the impacts on subsequent hysterectomy, if needed. Studies with longer follow-up durations are ongoing in order to answer these questions. In addition, we have advanced the search for new solutions with our laparoscopic long mesh surgery (LLMS) with augmented round ligaments.

Although sacrocolpopexy has been considered the gold-standard procedure for the treatment of apical prolapse, the long operation time and steep learning curve have restricted its popularity^4,5^. Additionally, most of the data have been related to post-hysterectomy prolapse^5^. Transvaginal mesh (TVM) implantation, once considered a breakthrough innovation in treating POP by experienced urogynecologists, has been criticized for the insufficient evidence supporting its safety^6^. Considering the abovementioned concerns, we attempt to invent a novel laparoscopic uterus-sparing procedure with synthetic mesh.

LLMS with augmented round ligaments was designed to create ventral uterine suspension by anchoring the mesh to the cervix and round ligaments. Our study aimed to assess whether this new procedure was effective, safe, and time-saving. In addition, the surgical complications and functional outcomes were also evaluated.

## Results

Of the 25 women undergoing LLMS with augmented round ligaments, 23 (92%) had a successful anatomic correction in the apical compartment without any major complications during the 12–24 month follow-up.

### The surgical outcome of POP

The baseline demographic data were obtained in the 25 women, including age, parity, body mass index (BMI), menopausal status, number under hormone therapy, underlying disease, baseline POP stage, concomitant procedures and follow-up period, as shown in Table 1. The mean age of our study population was 55.3 years old, and the mean parity was 1.9. Eleven (44%) women had stage 2 apical prolapse, and 14 (56%) had stage 3 apical prolapse. Fifty-two percent of women had prolapse in multiple compartments. Except for the unchanged total vaginal length (9 (9~11) vs. 9 (9~10), *p* = 0.52), other POP quantification (POP-Q) measurements before and after the surgeries all revealed statistically significant differences (Table 2). There were 2 (8%) cases of apical prolapse recurrence and 1 (4%) case of anterior compartment prolapse recurrence after surgery. One woman suffered from both anterior and apical prolapse, and the other woman had apical prolapse alone postoperatively; the original POP stages of these 2 women were stage 3 and 2, respectively.

**Table 1:** Demographic characteristics of the women who received laparoscopic long mesh surgery with augmented round ligaments (n = 25).

Parameters	mean± SD n (%)
Age (years)	55.3 ± 10.8
Parity	1.9 ± 0.5
BMI (kg/m^2^)	24.0 ± 3.2
Menopause	17 (68)
Current hormone therapy	1 (4)
Diabetes mellitus	1 (4)
Hypertension	8 (32)
Baseline apical stage II POP	11 (44)
with anterior stage II POP	4 (16)
with anterior stage III POP	3 (12)
with posterior stage II POP	1 (4)
Baseline apical stage III POP	14 (56)
with anterior stage III POP	3 (12)
with posterior stage II POP	2 (8)
Concomitant procedures in this study	
Anterior colporrhaphy	3 (12)
Posterior colporrhaphy	3 (12)
Cervical amputation	9 (36)
Myomectomy	2 (8)
Midurethral sling	3 (12)
Follow-up (months)	12–24

^†^Data are given as the mean ± standard deviation or n (%).

^‡^BMI: body mass index; POP: pelvic organ prolapse.

**Table 2:** Pelvic organ prolapse quantification (POP-Q) values before and after surgery.

POP-Q parameters	Preoperative (n = 25)	Postoperative (n = 25)	*p* value^*^
Aa	0 (−3~1.5)	−2 (−3~−1)	<0.001
Ba	2.5 (−0~3)	−2 (−3~−1)	<0.001
C	2.5 (−3~3)	−8 (−6~−10)	<0.001
Ap	−2 (−3~−2)	−3 (−3~−2)	0.030
Bp	1 (−3~2)	−2 (−3~−1)	0.002
TvL	9 (9~11)	9 (9~10)	0.521
Recurrent POP			
Apical prolapse		2 (8)	
Anterior vaginal prolapse		1 (4)	
Posterior vaginal prolapse		0	

^*^The Wilcoxon signed-rank test.

^†^Data are given as the median (range) or n (%).

### Improvements in symptoms, questionnaires, and urodynamic parameters

In terms of lower urinary tract symptoms (LUTS), many factors significantly improved after LLMS, including urinary frequency (36% vs. 4%, *p* = 0.021), stress urinary incontinence (SUI) (80% vs. 24%, *p* < 0.001), feeling of incomplete emptying (56% vs. 8%, *p* < 0.001), and hesitancy (52% vs. 16%, *p* = 0.004) (Table 3). The scores of the questionnaires, including the Overactive Bladder Symptom Score (OABSS) and the short forms of the Urogenital Distress Inventory (UDI-6), the Incontinence Impact Questionnaire (IIQ-7), and the Pelvic Organ Prolapse Distress Inventory (POPDI-6), all improved significantly after the operations, as shown in Table 3. The urodynamic study revealed that none of the participants had voiding or storage dysfunction after the surgeries; furthermore, the amount of residual urine decreased significantly in the urodynamic parameter analysis, as shown in Table 4. Of the 15 women who remained sexually active before the operation, the Female Sexual Function Index (FSFI) did not differ significantly for each item or in terms of the total scores pre- and post-operatively (Table 5).

**Table 3:** Urinary symptoms and quality of life questionnaires before and 6 months after surgery.

Symptoms	Preoperative (n = 25)	Postoperative (n = 25)	*p* value
Urinary frequency	9 (36)	1 (4)	0.021^***^
Stress urinary incontinence	20 (80)	6 (24)	<0.001^***^
Urgency urinary incontinence	6 (24)	2 (8)	0.22 ^***^
Feeling of incomplete bladder emptying	14 (56)	2 (8)	<0.001^***^
Hesitancy	13 (52)	4 (16)	0.004^***^
Nocturia	18 (72)	13 (52)	0.27 ^***^
OABSS	4.8 ± 3.3	3.9 ± 1.8	0.004^***^
UDI-6	25.1 ± 16.8	14.4 ± 6.8	0.009^***^
IIQ-7	32.4 ± 24.3	13.3 ± 12.8	<0.001^***^
POPDI-6	9.2 ± 4.8	5.5 ± 3.0	<0.001^***^

^*^McNemar’s test.

^**^Fisher’s exact test.

^***^Paired *t*-test.

^†^Data are given as the mean ± standard deviation or n (%).

^‡^Pre-op, preoperative; post-op, postoperative; OABSS, overactive bladder symptom score; UDI-6, Urogenital Distress Inventory; IIQ-7, the Incontinence Impact Questionnaire; POPDI-6, pelvic organ prolapse distress inventory 6.

**Table 4:** Urodynamic changes before and 6 months after surgery.

Parameters	Preoperative (n = 25)	Postoperative (n = 25)	*p* value
Qmax (mL/s)	19.4 ± 6.7	18.8 ± 4.0	0.71^**^
RU (mL)	31.0 ± 14.5	11.2 ± 5.7	0.006^**^
DO	8 (32)	3 (12)	0.70^**^
FS (mL)	157.7 ± 49.5	154.5 ± 62.3	0.63^**^
MCC (mL)	287.2 ± 79.4	311.0 ± 59.5	0.07^**^
PdetQmax (cmH_2_O)	24.5 ± 12.9	23.0 ± 15.9	0.52^**^
FUL (mm)	28.4 ± 5.4	29.4 ± 6.8	0.82^**^
MUCP (cmH_2_O)	52.3 ± 21.2	54.1 ± 20.7	0.75^**^

^*^Fisher’s exact test.

^**^Paired *t*-test.

^†^Data are given as the mean ± standard deviation or n (%).

^‡^Pre-op, preoperative; post-op, postoperative; Qmax, maximum flow rate; RU, residual urine; DO, detrusor overactivity; FS, first sensation to void; MCC, maximum cystometric capacity; PdetQmax, detrusor pressure at peak flow; FUL, functional urethral length; MUCP, maximum urethral closure pressure.

**Table 5:** Changes in scores of the Female Sexual Function Index before and 6 months after surgery.

Parameters	Preoperative (n = 15)	Postoperative (n = 15)	*p* value^*^
Sexual desire	3.5 ± 0.5	3.6 ± 0.8	0.77
Sexual arousal	3.9 ± 0.9	3.8 ± 0.6	0.48
Lubrication	4.5 ± 0.8	4.1 ± 1.3	0.27
Orgasm	4.7 ± 0.9	4.3 ± 0.5	0.12
Satisfaction	4.8 ± 0.9	4.9 ± 0.8	0.16
Dyspareunia	4.9 ± 1.3	4.1 ± 1.3	0.12
Total scores	26.3 ± 4.6	24.8 ± 3.0	0.19

^*^Paired *t*-test.

^†^Data are given as the mean ± standard deviation.

### Intraoperative and postoperative complications

The operative time of all women was 65.4 ± 28.8 minutes, and no intraoperative complications were noted (Table 6). The postoperative surgical wound pain was tolerable. Three of the 25 participants experienced urinary tract infection after surgery, and all of them were treated with oral cephradine (Unifradine^®^, Bora Pharmaceuticals, Tainan, Taiwan) for 7 days without any sequelae. No recurrence of urinary tract infection in these 3 women was reported during the follow-up period.

**Table 6:** Intraoperative and postoperative complications.

	n = 25	Clavien-Dindo Classification
Operative time (minutes)	65.4 ± 28.8	
Intraoperative complications		
Bladder injury	0	
Rectal injury	0	
Blood transfusion	0	
Conversion to open surgery	0	
Postoperative complications		
Postoperative day 1 VAS score	2.1 ± 1.5	Grade I
Urinary tract infection	3 (12)	Grade II
Voiding dysfunction	0	
Pelvic hematoma	0	

^†^Data are given as the mean ± standard deviation or n (%).

^‡^VAS, visual analogue scale.

## Discussion

LLMS with augmented round ligaments is a novel surgical method for apical prolapse with a high anatomical reduction rate, short operation time, and low complication rate in women with dominant apical prolapse who prefer uterine preservation in their POP surgeries. Concomitant anterior and/or posterior colporrhaphy may sometimes be needed in multi-compartment POP.

TVM surgeries have become popular in the last several years due to the favorable surgical outcomes and the easy approach, regardless of hysteropexy or hysterectomy surgeries^7,8^. However, there are public concerns regarding the safety of TVM since the long-term evidence is still insufficient. On April 16, 2019, the FDA ordered all manufacturers to stop selling synthetic mesh intended for transvaginal repair of anterior compartment prolapse because the manufacturers had not demonstrated a reasonable assurance of safety and effectiveness for these devices^6^. Therefore, native tissue repair and abdominal approach repair are expected to gain increasing attention for POP management because of the expected decreasing trend of TVM use. For apical prolapse, laparoscopic approaches, such as LLMS, are minimally invasive and have a lower reoperation rate than native tissue repair^9^.

In a Cochrane review, compared with a variety of vaginal interventions, sacrocolpopexy or sacrohysteropexy was demonstrated to be associated with a lower risk of subjective or objective prolapse recurrence, repeat surgery for prolapse, postoperative SUI and dyspareunia, serving as the gold standard for apical prolapse^5^. However, the benefits must be balanced against the long operating time and the need for advanced laparoscopic suturing skills in laparoscopic sacrocolpopexy^10,11^. In addition, the long-term risk of mesh or suture extrusion should also be of deep concern for sacrocolpopexy^12^, since mesh extrusion can sometimes lead to severe consequences^13^.

LLMS has the advantage of being a minimally invasive approach without the disadvantages associated with sacrocolpopexy, such as the long operation time and the possibility of severe complications. It results in a high success rate of anatomic reduction during a follow-up of 1–2 years. The dissection method of LLMS is similar to that of ventral suspension in pectopexy, which fixes the descending organ to the iliopectineal ligaments using polyvinylidene fluoride monofilament mesh^14–16^. In a systematic review by Szymczak P and colleagues, pectopexy showed similar high patient satisfaction as sacrocolpopexy (96.4–97.6% vs. 71.0–100%), although long-term follow-up results are still required^17^. However, the operation field of pectopexy is surrounded by external iliac vessels and the obturator nerve. Furthermore, fixation to the iliopectineal ligaments of the mesh is non-adjustable, either with single interrupted sutures or continuous sutures^18^.

In LLMS with augmented round ligaments, the operation field is relatively safe and easily approached for surgeons. Additionally, the mesh is pulled out of the skin from the abdominal wound medial to the anterior superior iliac spine (ASIS), providing an easy intraoperative tension adjustment mechanism, either tighter or looser. The uterus can reach an appropriate position with an adequate total vaginal length. Most importantly, the barbed sutures between the bilateral round ligaments and mesh can enhance tissue reaction and fibrosis, making the surgical outcome more robust. The round ligaments can also be folded to an ideal length during suturing if they are too long due to the stretching by descending organs, making tissue remodeling even better. This approach is very different from laparoscopic pectopexy or other types of lateral suspension using mesh, simply creating a new “mesh” ligament without augmentation of the original round ligament^17^.

Female sexual dysfunction is common among women with urogynecological conditions^19^ and should be managed with a multidisciplinary approach^20^. Tahaoglu AE and colleagues demonstrated that modified laparoscopic pectopexy improved the scores of the FSFI and Prolapse Quality of Life questionnaire^14^. Our study also showed a great improvement in LUTS and questionnaire scores regarding LUTS-related quality of life. However, the FSFI scores did not improve significantly after LLMS. The age of the population in Tahaoglu’s study was 39.09 ± 8.2 years old, which was much younger than our population (55.3 ± 10.8 years old) and probably suggests a confounding factor of menopause in our study. The pre-operative FSFI scores were much lower in their study than in our study but were similar postoperatively, suggesting a higher rate of sexual dysfunction in their population. Therefore, further studies comparing improvements in FSFI scores between sexual dysfunction and non-dysfunction groups may be needed to measure the changes in sexual function after LLMS.

The results regarding the urodynamic parameters revealed no voiding or storage function disturbances after surgery. The amount of residual urine also decreased significantly after successful anatomic reduction. LLMS significantly improved most of the LUTS, except for urgency urinary incontinence and nocturia. The urodynamic study results also revealed no significant decrease in the detrusor overactivity (DO) rate. The findings described above suggest that even though LLMS provides fair anatomical reduction, concomitant medical treatment is sometimes needed for the treatment of urgency urinary incontinence and nocturia in these patients. Women with urgency urinary incontinence, or overactive bladder (OAB)-wet, have a small bladder capacity and poor urethral closing mechanism and are usually treated with antimuscarinic agents^21^. Nocturia is a symptom typically associated with OAB^22^. Moreover, if women have nocturnal polyuria, desmopressin should be applied for better treatment efficacy^23^. Since OAB-wet and nocturia should be better managed with medication, as a surgical treatment for anatomical reduction, LLMS is insufficient for curing these LUTS.

The horizontal traction level between S1 and S2 in LLMS is similar to that in pectopexy. This anchor point is appropriate for the physiological axis of the vagina, and no de novo defecation symptoms were observed after pectopexy^15^ or after LLMS in our study. The mesh was embedded beneath the peritoneum and fixed onto the uterine cervix ventrally in our study, avoiding the major complications that occur in sacrocolpopexy^12,13^.

LLMS is a safe procedure, as the only complication observed in our study was UTI. There were 2 cases of recurrence in our study, occurring in the second and 5th cases. This can be explained by the fact that a learning curve still exists for this novel procedure. Due to the small number of cases of recurrence, our data are insufficient for addressing the patient characteristics regarding the risk of recurrence. For these 2 cases, we then further performed vaginal hysterectomy and sacrospinous ligament fixation to solve the problem of recurrent POP. Among all the cases of recurrent POP, vaginal vault prolapse was the most difficult to manage. Studies have provided strategies for prevention^24^ and novel methods of treatment^25^. All techniques have been suggested to be effective, but a comparison of the techniques is difficult because of heterogenicity^26^. LLMS is a uterine preservation POP surgery, and operators can still harvest the uterine ligaments for suspension or fixation for recurrent POP instead of managing vaginal vault prolapse.

The preliminary data for LLMS suggests that this procedure meets the needs of the increasing requests for uterine preservation, as it is a minimally invasive POP surgery with a high anatomical reduction rate, a short operation time, and few major intraoperative complications, although further studies with larger sample sizes should be conducted to make these results more robust. This procedure has the advantage of synthetic mesh durability while minimizing the concern for internal organ injury by being completely concealed under the peritoneum. It avoids the possible major complications associated with laparoscopic sacrocolpopexy; it solves the concerns regarding the seemingly compressed urinary bladder as well as subsequent hysterectomy concerns of LONG surgery. The study of long-term efficacy is ongoing and promising.

There is no doubt that the flaws of our study are the limited number of cases and the short follow-up period. In terms of mesh surgeries, a two-year follow-up sometimes cannot reveal all the complications and durability. Long-term follow-up results of stitch and mesh extrusion after sacrocolpopexy have been reported^12^. In addition to inadequate mesh material, the risk factors for mesh extrusion in sacrocolpopexy are concurrent hysterectomy and smoking^27^. In LLMS, the utero-vesical fold is dissected to expose the anterior colpo-cervical junction; thus, it is possible that stitch and mesh extrusion occurs in the bladder. However, due to the relatively safe operation field, we can expect a very low risk of severe complications, such as bowel perforation, bladder perforation, and vessel injury, in further follow-up studies. Additionally, the surgical outcomes should be robust after a longer follow-up due to the extensive fibrosis of long tracts of tissue (the cervix and bilateral round ligaments). LLMS is suitable for surgeons of any skill level. A study with a larger sample size and a longer follow-up for LLMS should be conducted in the future.

## Patients and Methods

**Figure 1:**
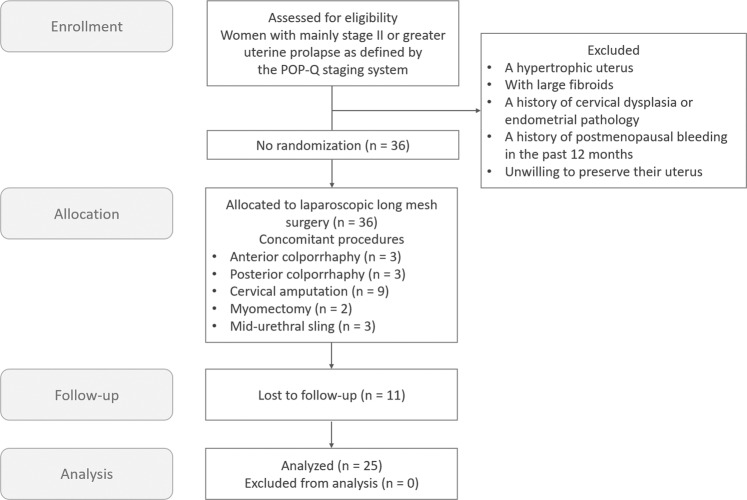
The clinical trial flowchart for laparoscopic long mesh surgery with augmented round ligaments.

From August 2017 through November 2018, 36 consecutive women with mainly stage II or greater uterine prolapse as defined by the POP-Q staging system^28^ were referred for LLMS with augmented round ligaments at a tertiary referral center in Taiwan. We excluded women with a hypertrophic uterus, large fibroids (sonographic size of more than 10 cm in diameter), a history of cervical dysplasia or endometrial pathology, a history of postmenopausal bleeding in the past 12 months, and those unwilling to preserve their uterus before enrollment into study. Concomitant mid-urethral sling operations were performed in women with current urodynamic stress incontinence unless they did not prefer a concomitant surgery. These mid-urethral sling procedures included MiniArc (AMS, Inc., Minnetonka, MN, USA) and TVT-O (Gynecare TVT-Obturator System, Ethicon, Inc., Somerville, NJ). Cervical amputation was performed if the corpus/cervix ratio was less than 1 on the ultrasound^29^. Concomitant anterior and posterior colporrhaphy procedures were performed as needed. Myomectomy was performed under certain indications, such as hypermenorrhea, dysmenorrhea, mass-effect-related constipation or urinary frequency, or a fast-growing mass. Eleven women with incomplete medical records or who were lost of follow-up were also excluded. Ultimately, this cohort study was conducted on the basis of 25 available subjects (Fig. 1).

### Operative technique: Laparoscopic long mesh surgery

**Figure 2:**
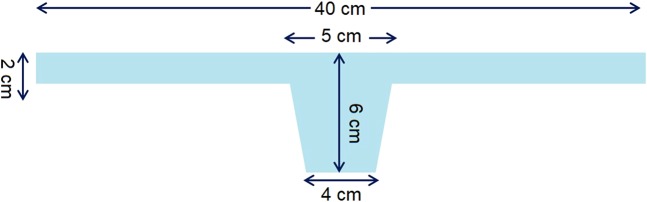
The parameters and design of the long mesh.

**Figure 3:**
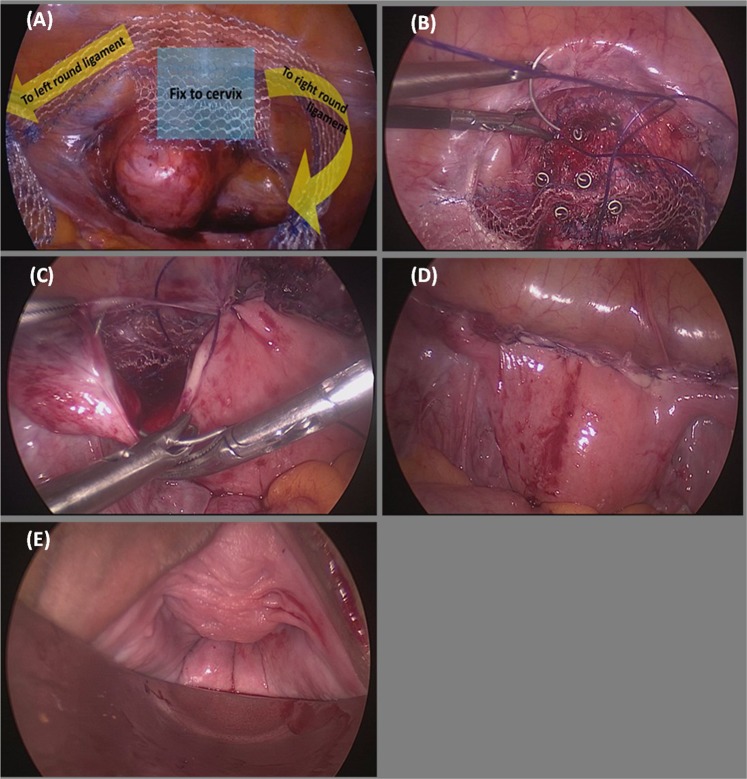
The procedural process. (**A**) Under laparoscopy, the peritoneum of the utero-vesical fold was dissected to expose the anterior colpo-cervical junction. T-shaped self-tailored long mesh was delivered to the operation field. Bilateral mesh legs were extracted outside the trocar wounds to stabilize the mesh position. (**B**) Center piece of the mesh was fixed to the cervix with ProTack screws (Covidien, New Haven, Connecticut), Stratafix 2-0 sutures (Ethicon, Norderstedt, Germany) and Tisseel fibrin sealant (Baxter, Deerfield Illinois). (**C**) Bilateral round ligaments and the mesh arms were sutured continuously with Stratafix 2-0. (**D**) Reperitonealization with the remaining Stratafix 2-0 sutures. (**E**) The tension of the mesh was adjusted until the cervix was reduced to the deepest point according to the vaginal examination.

All surgical procedures were performed under general anesthesia. Every patient received a single dose of intravenous prophylactic antibiotics. The patients were placed in a lithotomy position. Laparoscopy was set up with the endoscope located at the 10-mm umbilical wound, two 5-mm trocar ports in the bilateral lower quadrant of the abdomen (2 cm medial to the anterior superior iliac spine), and one 5-mm trocar port 8 cm left and lateral to the umbilicus. The peritoneum at the utero-vesical fold was dissected to expose the anterior colpo-cervical junction. Long mesh, a synthetic T-shaped mesh (Fig. 2, Gynemsh, Ethicon, San Lorenzo, Puerto Rico), was delivered into the pelvic cavity (Fig. 3A). Bilateral mesh arms were extracted outside the trocar wounds bilaterally to stabilize the mesh position. The center piece was fixed to the cervix with 5-mm ProTack screws (Covidien, New Haven, Connecticut) (Fig. 3B). Fixation was strengthened with Stratafix 2–0 sutures (Ethicon, Norderstedt, Germany) (Fig. 3B), followed by Tisseel fibrin sealant (Baxter, Deerfield, Illinois) for better hemostasis among the surrounding tissues. An extraperitoneal tunnel was created along the left round ligament until it reached a location 2 cm medial to ASIS. One arm of the long mesh was pulled out along the tunnel underneath the round ligament and fixed with the fascia of the abdominal oblique muscle. The same procedure was repeated on the contralateral side. The bilateral round ligaments and the mesh arms were sutured continuously with Stratafix 2–0 (Fig. 3C). Reperitonealization was carried out thereafter (Fig. 3D). The tension of the mesh was adjusted until the apical compartment was reduced to an appropriate position per the vaginal examination (Fig. 3E).

The baseline demographic data included age, parity, body mass index (BMI), menopausal status, number under hormone therapy, underlying disease, baseline POP stage, concomitant procedures and follow-up period. The clinical evaluations consisted of a detailed history before and 6 months after surgery, including urinary analysis, pelvic examination using the POP-Q system^22^, urodynamic study (UDS), transabdominal ultrasound, and personal interview to identify urinary and sexual symptoms with the Overactive Bladder Symptom Score (OABSS)^30^ and the short forms of the Urogenital Distress Inventory (UDI-6), the Incontinence Impact Questionnaire (IIQ-7)^31^, the Female Sexual Function Index (FSFI) questionnaire^32^, and the Pelvic Organ Prolapse Distress Inventory (POPDI-6)^33^. Urinary symptoms were assessed with the standardized questionnaire taking into account the 2002 ICS definitions^34^. Women were asked to fill out visual analog scale (VAS) scores during rounds on postoperative day 1. Urodynamic studies, including non-instrumented uroflowmetry, filling and voiding cystometry, and urethral pressure profilometry, were performed according to the recommendations of the International Continence Society^35^ with a 6-channel urodynamic monitor (MMS; UD2000, Enschede, Netherlands). Any uninhibited detrusor contraction during filling cystometry was deemed positive for detrusor overactivity (DO).

The primary outcome of this study was the degree of anatomical reduction after the surgery as assessed by pelvic examination using the POP-Q system. Regarding the secondary outcomes, LUTS and sexual function were assessed with the questionnaires listed above, and storage and voiding function was evaluated by UDS 6 months after the surgery. For follow-up, postoperative outpatient visits were conducted at 1, 2, 3, 6, and 12 months and then semiannually beyond one year. A pelvic examination was performed routinely during every visit to the clinic. Recurrence was defined as the most dependent portion of stage II or greater POP in the anterior, apical and posterior compartments. Clavien-Dindo grading was used for the classification of the intraoperative and postoperative complications of LLMS^36^.

We assessed the power of tests for differentiating the surgical outcomes of the LLMS procedure, and power analysis showed that approximately 25–30 women in this study would have a power of 80% and a 5% significance level in this one-tail test. Expecting a withdrawal rate of 20%, we enrolled a total of 36 women for this study. Although some comparisons, such as the rate of DO, could not reach sufficient power due to the limited number of patients, we utilized multiple parameters of the POP-Q system to evaluate postoperative changes. We found that for our 24 female subjects, there would be a power of over 82% for discrimination.

### Statistics

IBM SPSS Statistical Software version 20.0 ed. was used for the statistical analyses. The Wilcoxon signed-rank test was performed for comparison between preoperative and postoperative POP-Q parameters. McNemar’s and Fisher’s exact tests were performed for categorical variables. Paired *t*-tests were performed for two related units on a continuous outcome. A *p*-value of less than 0.05 was considered statistically significant.

### Ethical approval and clinicaltrials registration

This study received approval from the Institutional Review Board of Kaohsiung Medical University Hospital (ID: KMUHIRB-E(I)-20190015), by which relevant guidelines and regulations were followed accordingly. This study was also registered at ClinicalTrials.gov (ID: NCT04139083, registered on 25/10/2019).

### Informed consent

Informed consent was obtained from all participants before the surgeries.

## Conclusions

LLMS with augmented round ligaments is a novel surgical method for women with apical prolapse and a desire for uterine preservation. It provides not only anatomic correction of POP but also improvements in LUTS and quality of life. The short-term follow-up results of the surgery showed it has a high efficacy, is time-saving, and has a relatively low complication rate for women with main apical prolapse. Concomitant anterior and/or posterior colporrhaphy may sometimes be needed in multi-compartment POP.

## Supplementary information

Clinical Study Protocol.

## Data Availability

The datasets analyzed during the current study are available from the corresponding author upon reasonable request.
