# Supplementary material for: Laparoscopic Long Mesh Surgery with Augmented Round Ligaments: A Novel Uterine Preservation Procedure For Apical Pelvic Organ Prolapse
Source: Sci Rep. 2020 Apr 20;10:6615. doi: 10.1038/s41598-020-63725-x (PMC7171133; doi:10.1038/s41598-020-63725-x)
Supplement: Supplementary file 1 — Clinical Study Protocol. [file 41598_2020_63725_MOESM1_ESM.docx]

**Clinical Study Protocol**

The Clinical and Urodynamic Effect

of Transvaginal or Laparoscopic mesh suspension

for The Treatment of Pelvic Organ Prolapse

|  |  |
| --- | --- |
| Version: | 1.0 |
| Date: | 05 January 2019 |
|  |  |
|  |  |

| PI | Cheng-Yu Long |
| --- | --- |
| Co-PI | Kun-Ling Lin |

*The original version of this clinical study protocol was written in Mandarin. This is an English version according to the request of the editorial office. The formal IRB-proved version can also be provided if necessary. Our original article is part of this clinical study protocol.

# Background

Female pelvic floor dysfunction (PFD) includes symptoms of urinary incontinence (UI), anorectal dysfunction, pelvic organ prolapse (POP), sexual dysfunction, pelvic pain, and lower urinary tract abnormalities. High recurrence rates were noted over traditional pelvic floor reconstruction. However, the safety and efficacy of transvaginal mesh and laparoscopic mesh suspension are still undetermined.

# Study Objectives

To evaluate the clinical and urodynamic effect of transvaginal mesh and laparoscopic mesh suspension for the treatment of pelvic organ prolapse.

# Study Design and Center

## Study Period: January 1^st^, 2012 to December 31^st^, 2018.

## Location: Kaohsiung Medical University Chung-Ho Memorial Hospital

### Inclusion Criteria

Women with mainly uterine prolapse stage II or greater as defined by the POP-Q staging system received transvaginal mesh surgeries and laparoscopic mesh suspension surgeries.

### Exclusion Criteria

- Age < 20-year-old
- A hypertrophic uterus, huge fibroids
- History of cervical dysplasia or endometrial pathology
- History of postmenopausal bleeding in the past 12 months
- Unwilling to preserve their uterus

# Methodology

## Patients’ data

The clinical evaluations consisted of a detailed history before and 6 months after surgery, including urinary analysis, pelvic examination using POP-Q system, urodynamic studies, transabdominal ultrasound, and personal interview to identify urinary and sexual symptoms. Urinary symptoms with the standardized questionnaire taking into account the 2002 ICS definitions. Women were asked to fill out the visual analog scale (VAS) scores during the postoperative day 1 round.

As a follow-up, postoperative outpatient visits were at 1, 2, 3, 6, and 12 months and then semiannually beyond one year. Pelvic examination was performed routinely in every visit to clinics. Recurrence was defined as the most dependent portion of POP stage II or greater. The Clavien-Dindo grading was used for the classification of the intraoperative and postoperative complications of Long mesh surgery.

## Equipment

Urodynamic studies, including non-instrumented uroflowmetry, filling and voiding cystometry, and urethral pressure profilometry, were performed according to the recommendations by the International Continence Society with a 6-channel urodynamic monitor (MMS; UD2000, Enschede, Netherlands).

## Questionnaires

To identify urinary and sexual symptoms with the Overactive Bladder Symptom Score (OABSS), the short forms of Urogenital Distress Inventory (UDI-6), the Incontinence Impact Questionnaire (IIQ-7), the Female Sexual Function Index (FSFI) questionnaire, and the Pelvic Organ Prolapse Distress Inventory (POPDI-6).

# Statistical Analysis

Retrospective and observational design. IBM SPSS Statistical Software version 20.0 ed. was used for statistical analyses.

# Expected results

- 1. Evaluate the clinical outcomes of unabsorbable mesh in pelvic reconstruction surgeries.
  2. Compare the urodynamic parameter changes after pelvic reconstruction surgeries.
  3. Compare the changes in the lower urinary tract symptoms and the sexual function after different surgeries.
  4. Provide information for health caregiver in pre-operative evaluation.

# study stAff

| PI | Cheng-Yu Long | Professor |
| --- | --- | --- |
| Co-PI | Kun-Ling Lin | Assistant professor |

# REFERENCES

1. Haylen BT, de Ridder D, Freeman RM, et al. An International Urogynecological Association (IUGA)/ International Continence Society (ICS) joint report on the terminology for female pelvic floor dysfunction. Int Urogynecol J 2010;21: 5–26.
2. Nygaard I, Barber MD, Burgio KL, et al. Prevalence of symptomatic pelvic floor disorders in US women. JAMA 2008; 300(11):1311–6.
3. Olsen AL, Smith VJ, Bergstrom JO, Colling JC, Clark AL. Epidemiology of surgically managed pelvic organ prolapse and urinary incontinence. Obstet Gynecol 1997;89:501-6.
